# Cytoglobin promotes sensitivity to ferroptosis by regulating p53‐YAP1 axis in colon cancer cells

**DOI:** 10.1111/jcmm.16400

**Published:** 2021-02-21

**Authors:** Shazhou Ye, Mingjun Xu, Tingwei Zhu, Jiayi Chen, Shanping Shi, Haizhong Jiang, Qingfang Zheng, Qi Liao, Xiaoyun Ding, Yang Xi

**Affiliations:** ^1^ Institute of Biochemistry and Molecular Biology School of Medicine Ningbo University Ningbo China; ^2^ Department of Gastroenterology Ningbo First Hospital Ningbo China; ^3^ Department of Preventative Medicine School of Medicine Ningbo University Ningbo China

**Keywords:** colon cancer, cytoglobin, ferroptosis, lipid peroxidation, p53, YAP1

## Abstract

Ferroptosis is an iron‐dependent mode of non‐apoptotic cell death characterized by accumulation of lipid reactive oxygen species (ROS). As a regulator of ROS, cytoglobin (CYGB) plays an important role in oxygen homeostasis and acts as a tumour suppressor. However, the mechanism by which CYGB regulates cell death is largely unknown. Here, we show that CYGB overexpression increased ROS accumulation and disrupted mitochondrial function as determined by the oxygen consumption rate and membrane potential. Importantly, ferroptotic features with accumulated lipid ROS and malondialdehyde were observed in CYGB‐overexpressing colorectal cancer cells. Moreover, CYGB significantly increased the sensitivity of cancer cells to RSL3‐ and erastin‐induced ferroptotic cell death. Mechanically, both YAP1 and p53 were significantly increased based on the RNA sequencing. The knock‐down of YAP1 alleviated production of lipid ROS and sensitivity to ferroptosis in CYGB overexpressed cells. Furthermore, YAP1 was identified to be inhibited by p53 knock‐down. Finally, high expression level of CYGB had the close correlation with key genes YAP1 and ACSL4 in ferroptosis pathway in colon cancer based on analysis from TCGA data. Collectively, our results demonstrated a novel tumour suppressor role of CYGB through p53‐YAP1 axis in regulating ferroptosis and suggested a potential therapeutic approach for colon cancer.

## 1 INTRODUCTION

Ferroptosis is a newly discovered mode of non‐apoptotic cell death that occurs as a consequence of increased lipid peroxidation due to the homeostatic turnover by accumulation of polyunsaturated fatty acid phospholipids, glutamate, iron or by depletion of antioxidant glutathione (γ‐L‐glutamyl‐L‐cysteinylglycine, GSH), NADPH, glutathione peroxidase 4 (GPX4).^1^, ^2^ Iron and iron derivatives are essential for the function of lipid peroxides. Ferroptotic cell death can be suppressed by iron chelators, lipophilic antioxidants, lipid peroxidation inhibitors and polyunsaturated fatty acid depletion.^3^ Ras selective lethal 3 (RSL3) and eradicator of Ras and ST (erastin) are two widely accepted ferroptosis‐inducing compounds that inactivate cellular glutathione (GSH)‐dependent antioxidant defences and act in an apoptosis‐independent manner.^4^ Specifically, RSL3 directly inhibits the activity of glutathione peroxidase 4 (GPX4), which eliminates lipid peroxides,^5^ while erastin blocks the glutamate/cystine antiporter of system xc‐ to import cysteine, which is a precursor of cellular GSH synthesis, and leads to a reduced level of GSH and ROS accumulation.^6^, ^7^

As a regulator of ROS, CYGB was originally discovered in rat hepatic stellate cells (HSCs) and is also named stellate cell activation‐associated protein (STAP).^8^, ^9^ Because the protein contains haem, a coordination complex of a porphyrin ring with an iron ion, this protein was renamed cytoglobin.^10^ CYGB is ubiquitously expressed in the mesenchymal fibroblastic cells of many organs, including the brain, liver and intestine. Because haem iron has demonstrated affinities for exogenous ligands and equilibrium constants for O2 that are similar to those observed in myoglobin, CYGB exhibits intrinsic oxygen (O2)‐binding capacity. Regarding its distribution in fibroblast‐like cells, which are not generally associated with high metabolic rates and oxygen consumption, CYGB might act as an oxygen sensor and be involved in cell proliferation and possibly oxygen diffusion for collagen synthesis.^9^ Multiorgan abnormalities, including tumours in the lung, liver and intestine, have been observed in *Cygb*‐deficient mice.^11^, ^12^ When subjected to treatment with N,N‐diethylnitrosamine (DEN), a liver‐specific carcinogen and fed a high‐fat diet, mice harbouring a hetero‐ or homozygotic deletion of *Cygb* were found to exhibit increased sensitivity to the drug, as all the mice developed liver tumours six months after the initiation of the DEN treatment.^13^ These reports suggest that CYGB is a tumour suppressor and that the loss of CYGB promotes sensitivity to tumorigenesis.^14^ It was further suggested that the promoter of *CYGB* was methylated, causing the silencing of *CYGB* expression, and that the restoration of CYGB could inhibit cancer cell growth.^15^ However, the mechanism by which CYGB regulates cell death is still largely unknown.

Recent discoveries have tried to reveal connections between ferroptosis and neoplastic diseases.^416^ Yes‐associated protein 1 (YAP1), the main effector of the Hippo signalling pathway, has been reported to regulate multiple biological processes, such as metabolism, tumorigenesis and ferroptosis.^17^, ^18^, ^19^ YAP1 can be phosphorylated by large tumour suppressor kinase 1/2 (LATS1/2) and degraded by proteasomes.^20^ When the Hippo/LATS1/2 pathway is turned off, YAP1 can shuttle into the nucleus and promote multiple transcriptional programs. YAP1 might act either as an oncogene or tumour suppressor depending on its binding partner and its subcellular localization.^21^ Indeed, YAP1 can function as an oncogene through its interactions with TEA domain transcription factors (TEAD) and is frequently amplified or hyperactivated in a number of human solid tumours.^22^ On the other hand, YAP1 has been reported to function as a tumour suppressor in breast cancer and haematological malignancies by promoting apoptosis in these contexts.^23^, ^24^ Moreover, a recent study demonstrated that YAP1 can promote ferroptosis.^25^

In the present study, we demonstrated that CYGB inhibits colorectal cancer (CRC) cell growth and promotes lipid peroxidation. Importantly, CYGB‐overexpressing cells are more sensitive to RSL3‐ and erastin‐induced ferroptosis. We also performed a mechanical demonstration that YAP1 is a key downstream target. Our results provide important molecular evidence of CYGB function.

## 2 MATERIALS AND METHODS

### 2.1 Cell culture and treatments

HCT116 human CRC cells were cultured in RPMI 1640 medium, and SW620 cells were maintained in DMEM with high glucose supplemented with 10% (v/v) foetal bovine serum and 100 U/ml penicillin‐streptomycin (Sigma‐Aldrich, USA). The short tandem repeat (STR) profiling of both cell lines was certificated at Shanghai Novobio Biotechnology (Shanghai, China). N‐Acetyl‐L‐cysteine (NAC, HY‐B0215), ferrostatin‐1 (Fer‐1, HY‐100579), (1S,3R)‐RSL3 (RSL3, HY‐100218A) and erastin (HY‐15763) were purchased from MedChemExpress (MCE) (Shanghai, China). Z‐VAD‐FMK (Z‐VAD, S7023) was purchased from Selleck Chemicals Company (Shanghai, China). RSL3 and erastin were applied to induce ferroptosis. Fer‐1 and NAC were used to inhibit ferroptosis. The concentrations are indicated in the text.

### 2.2 Cell proliferation and colony formation assay

Thiazolyl Blue (MCE, China) was used for the cell proliferation assay according to the manufacturer's protocol. The cells were plated in 96‐well plates, and the absorbance at 490 nm was measured. The relative viability normalized to the untreated condition was calculated.

For the cell colony formation assay, cells were seeded in 6‐well plates. After culturing for 2 weeks, the cells were fixed with 4% PFA and stained with 0.5% crystal violet solution (Solarbio, USA) at room temperature. Then, the cell colonies were counted and imaged.

### 2.3 Cell cycle and cell death analysis

Resuspended cells were incubated with 500 µL DNA staining solution and 10 µL permeabilization solution (MultiSciences, China) following the manufacturer's instructions. The DNA content was determined using flow cytometry (CytoFLEX S, Beckman).

Cell death was determined by propidium iodide (PI, BD, USA) staining. In brief, the cells were treated with test compounds for the indicated times, trypsinized, washed twice and resuspended in 500 µL PBS containing PI (500 ng/ml). After 20 minutes of incubation, the cell viability was detected using a flow cytometer.

### 2.4 Measurement of ROS and lipid peroxidation

The cellular or mitochondrial ROS levels were measured by staining with DCFH‐DA (25 µM, Sigma) or MitoSOX^TM^ Red (5 µM, Invitrogen) for 30 minutes at 37°C. The samples were then assessed using a flow cytometer.

To analyse lipid peroxidation, the cells were stained with 2 μM BODIPY‐C11 (Invitrogen) or assessed by measuring the malondialdehyde (MDA) levels using a lipid peroxidation MDA assay kit (Beyotime, China) according to the manufacturer's instructions.

### 2.5 Mitochondrial integrity and respiration measurements

The mitochondrial integrity was assessed by measuring the mitochondrial membrane potential (Δψ) using the fluorescent dye JC‐1 (BD, USA) according to the manufacturer's protocol. The samples were analysed using flow cytometry.

Real‐time monitoring of the cellular oxygen consumption rate (OCR) was performed using an XF96 extracellular flux analyser (Seahorse Bioscience) following the manufacturer's instructions. During the measurement, the following inhibitors of the respiratory chain components were serially added to the culture medium: the ATP synthase inhibitor oligomycin (1 µM); the respiratory uncoupler FCCP (0.5 µM); and the complex I and III inhibitors rotenone (0.5 µM) and antimycin A (0.5 µM).

### 2.6 Lentivirus, siRNAs, and transfection

To produce constitutive expression, a lentivirus carrying the CYGB CDS (protein coding region) fused with FLAG was constructed by and purchased from GeneChem Biotechnology (Shanghai, China). For CYGB overexpression, CYGB or MOCK viruses were applied to cells at an MOI of 20, and a two‐week drug selection with puromycin was performed to obtain stable cell lines.

The sequences of the YAP1 siRNAs were siYAP1‐1:5′‐GACAUCUUCUGGUCAGAGATT‐3′^26^ and siYAP1‐2:5′‐CCACCAAGCUAGAUAAAGATT‐3′,^27^ p53 siRNA was sip53: 5′‐GAGGUUGGCUCUGACUGUATT‐3′^28^ as reported by previous studies. The siRNAs were synthesized by GenePharma Biotechnology (Shanghai, China). The Lipofectamine® RNAiMAX Transfection Reagent (Invitrogen, Germany) was used for the knock‐down experiment following the manufacturer's instructions.

### 2.7 RNA extraction and qRT‐PCR

Total RNA was prepared with the TRIzol method (Invitrogen, Germany) and reverse transcribed using the GoScript Reverse Transcription (RT) System (Promega, USA). Quantitative RT‐PCR was performed using LightCycler 480 SYBR Green I Master on a LightCycler 480 II instrument (Roche, USA). The primers were as follows: CYGB‐F: 5′‐AGGCGAGATGGAGATCGAG‐3′; CYGB‐R: 5′‐ CTGGCTGAAGTACTGCTTGG‐3′; p53‐F: 5′‐GAGGTTGGCTCTGACTGTACC‐3′; p53‐R: 5′‐TCCGTCCCAGTAGATTACCAC‐3′; YAP1‐F: 5′‐ACAGCTCAGCATCTTCGACA‐3′; YAP1‐R: 5′‐TATTCTGCTGCACTGGTGGA‐3′; SLC7A11‐F: 5′‐GTTGCGTCTCGAGAGGGTCA‐3′; SLC7A11‐R: 5′‐GTCGAGGTCTCCAGAGAAGAGC‐3′; β‐actin‐F: 5′‐GCCGATCCACACGGAGTACTT‐3′; and β‐actin‐R: 5′‐TTGCCGACAGGATGCAGAA‐3′. The gene expression was normalized to that of β‐actin, and the relative fold change was calculated.

### 2.8 RNA sequencing

Extracted total RNAs were assessed with Agilent 2100 BioAnalyzer (Agilent Technologies, Santa Clara, CA, USA) and Qubit Fluorometer (Invitrogen). Total RNA samples that meet the following requirements were used in subsequent experiments: RNA integrity number (RIN) > 7.0 and a 28S:18S ratio > 1.8. Sequence libraries were generated with the NEB Next Ultra RNA Library Prep Kit for Illumina (NEB) and sequenced by CapitalBio Technology (Beijing, China). The triplicate samples of all assays were constructed an independent library.

High‐throughput RNA sequencing (RNA‐seq) was performed with pair end 150‐base pair reading length on an Illumina NovaSeq 6000 sequencer (Illumina, San Diego, CA) by CapitalBio Corporation (Beijing, China). Genes with a p‐value <= 0.01 and an expression ratio >= 2 or expression ratio <= 0.5 were recognized as significantly differentially expressed genes (DGEs) in the RNA‐seq analysis. The Kyoto Encyclopedia of Genes and Genomes database (KEGG) pathway enrichment analysis was performed for the DGEs using the clusterProfiler R package^29^ and KOBAS 3.0 software (available online: http://kobas.cbi.pku.edu.cn). Pathway terms with *P*‐value (or Q‐value) less than 0.05 were considered significantly enriched by target genes. RNA‐seq data sets have been deposited in GEO (Gene expression omnibus) under the accession number #GSE149426.

### 2.9 Fluorescence staining of cellular iron

FerroOrange (F374, Dojindo Molecular Technologies Inc) was used to detect the cellular iron. Briefly, HCT116 ‐MOCK and ‐CYGB cells were seeded on microscope cover glass in culture dishes for 24 hours and stained with FerroOrange (0.5 μM) for 30 minutes, washed and then observed under an inverted confocal laser scanning microscope equipped with a 60 × oil immersion optic (Leica, TCS SP8).

### 2.10 Western blot analysis

The proteins were isolated and separated by SDS‐PAGE. Primary antibodies against CYGB (#60228, Proteintech), YAP1 (#14074, Cell Signaling Technology), phospho‐YAP1 (Ser127) (#13008, Cell Signaling Technology), p53 (#2524, Cell Signaling Technology), phospho‐p53 (Ser15) (#9284, Cell Signaling Technology), SLC7A11 (#12691, Cell Signaling Technology), ACSL4 (#ab155282, Abcam) and GAPDH (#AP0063, Bioworld Antibodies) were used.

### 2.11 Statistical analysis

The data are presented as the mean ± standard deviation (SD). The statistical tests were performed with Prism GraphPad software. The Cancer Genome Atlas (TCGA) and the Genotype‐Tissue Expression (GTEx), and the Cancer Cell Line Encyclopedia (CCLE) data of mRNA expression were downloaded from the University of California Santa Cruz (UCSC) Xena website (https://xenabrowser.net/datapages/).^30^ We applied ‘limma’ package in R to screen differentially expressed genes. Here, we set |log2(Fold change)| > 2 and false discovery rate (FDR) < 0.05 as the cut‐off.^31^ Here, to further identify the role of CYGB, we used ‘clusterProfiler’ package in R to visualize the correlation.^29^ The Pearson correlation coefficient was used to assess the relationship between the two variables. Unless otherwise noted, p values were calculated using unpaired, two‐tailed t tests assuming unequal variance.

## 3 RESULTS

### 3.1 CYGB inhibited cell growth in colon cancer cells

**FIGURE 1:**
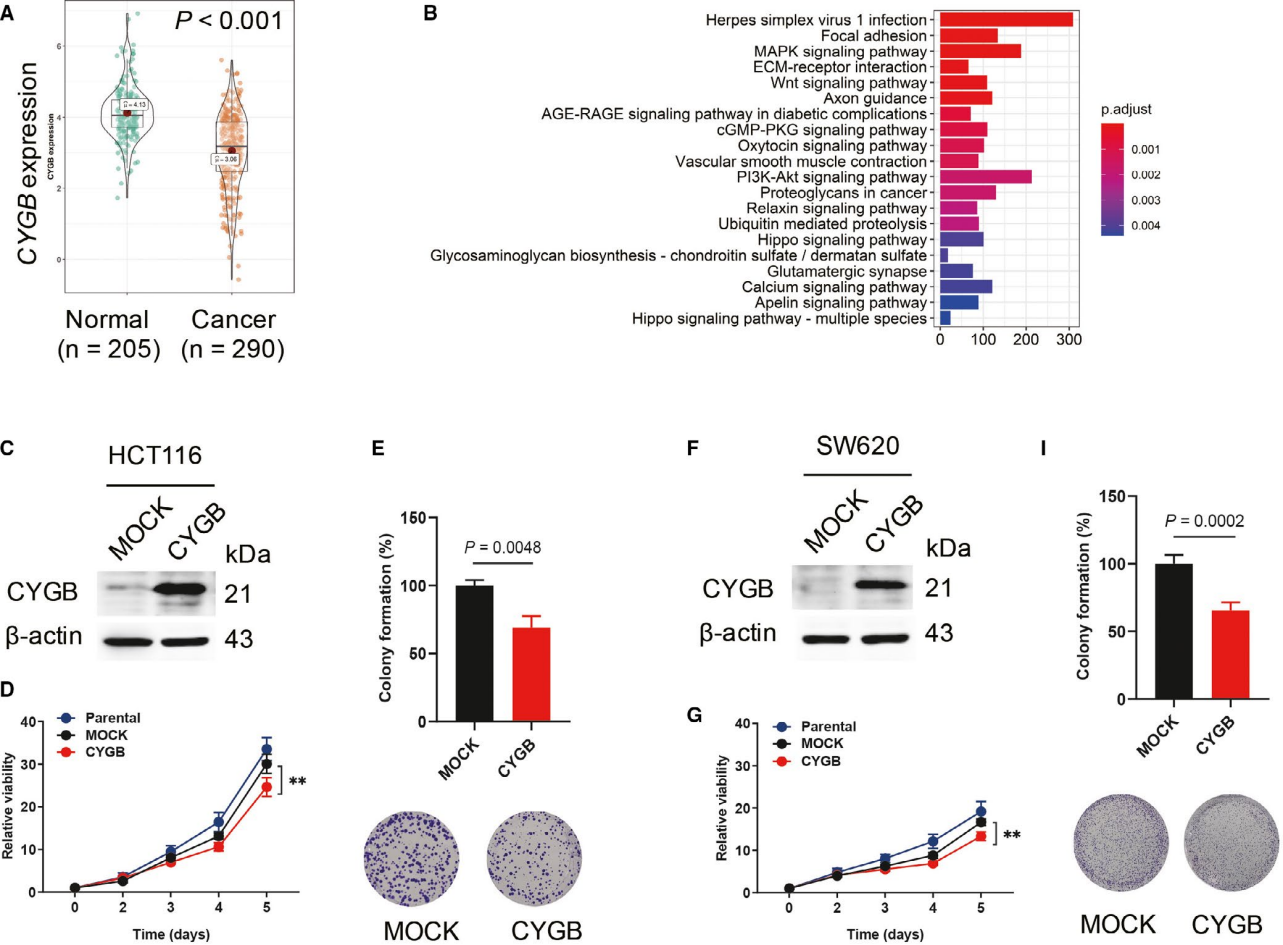
CYGB inhibited cell growth in colon cancer cells. A. Comparison of CYGB mRNA expression (transcripts per million in log scale) in COAD from the TCGA and GTEx databases. B. The list of significantly changed signalling pathways between comparison of CYGB‐low and CYGB‐high expression. The differentially expressed gens were collected based on the CYGB expression by the median expression value of *CYGB*. C, F. Confirmation of CYGB overexpression by immunoblotting in the human CRC cell lines HCT116 (C) and SW620 (F). D, G. Effects of CYGB on cell proliferation. Cell growth was determined on days 0, 2, 3, 4, and 5 in the CYGB‐overexpressing HCT116 (D) and SW620 cells (G). Relative viability normalized to the untreated condition was used. E, I. Colony formation assay. Single cells were cultured for eleven days to obtain colony formation and then stained with crystal violet solution

The TCGA (https://portal.gdc.cancer.gov/) and GTEx (https://www.gtexportal.org/home/index.html) databases were utilized to investigate the expression of *CYGB* in colon cancer and significantly decreased *CYGB* expression was observed in cancer tissues compared with normal tissues (*P* < 0.001) (Figure 1A). To further explore the function of CYGB, the CYGB expression was divided into CYGB‐low (CYGB‐L) and CYGB‐high (CYGB‐H) groups by the median expression value of *CYGB* to collect differentially expressed genes for functional pathway analysis. The top twenty significantly enriched KEGG pathways were listed in Figure 1B. Among them, Wnt and Hippo signalling pathways, which were involved in the cell growth, were included suggesting CYGB played regulatory role in cell growth. To further demonstrate the cellular function, CYGB lentivirus was applied to infect two well‐established cell lines HCT116 and SW620, and cells that constitutively expressed CYGB were obtained (Figure 1C,F). Significant inhibition of cell growth (Figure 1D,G) and single‐cell colony formation (Figure 1E,I) were confirmed in both the CYGB‐overexpressing cell lines. These above results demonstrated that CYGB inhibited cell growth in colon cancer cells.

### 3.2 CYGB promoted ROS production and disrupted mitochondrial function

**FIGURE 2:**
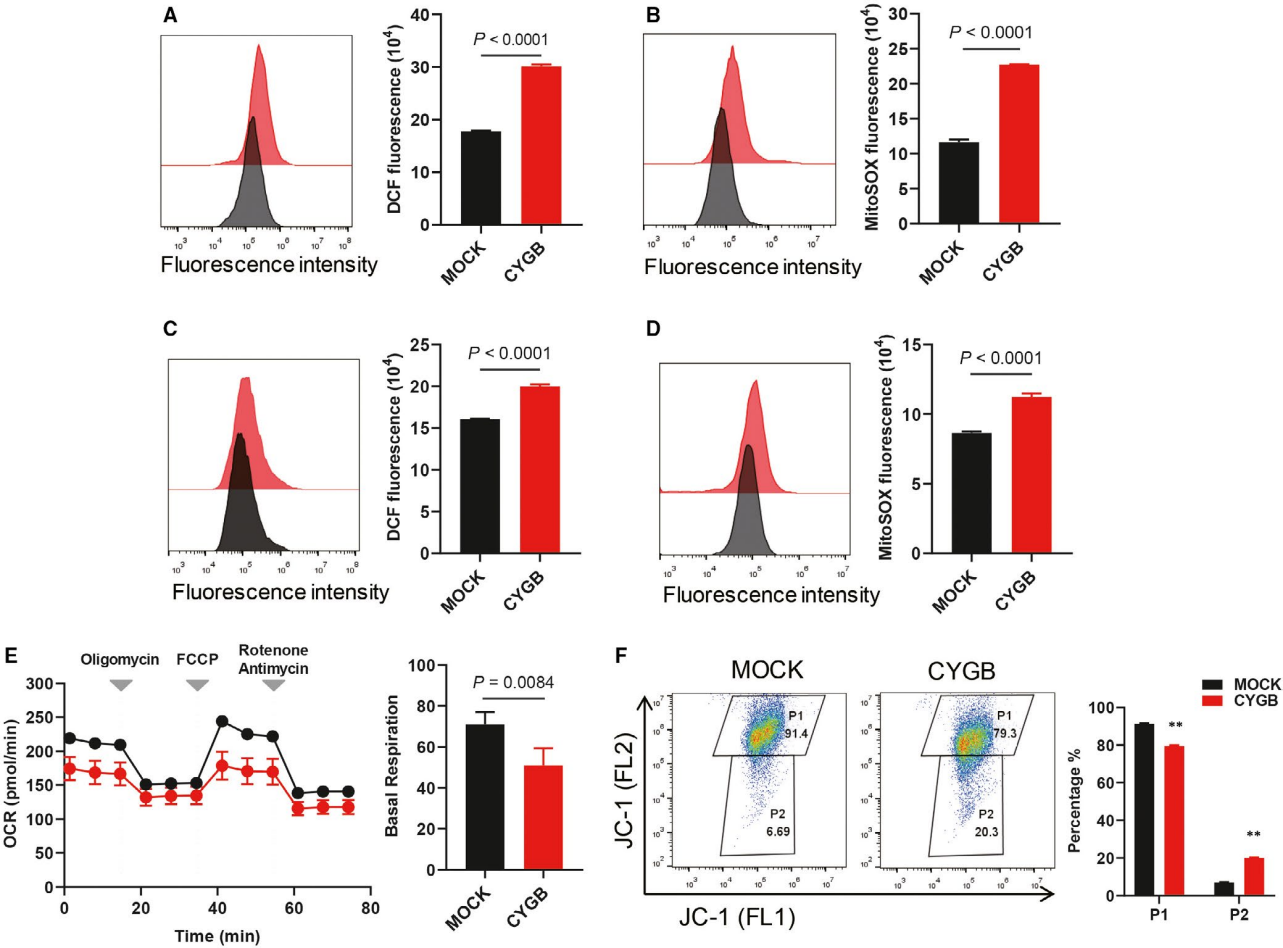
CYGB promoted ROS production and disrupted mitochondrial function. A, C. Cellular ROS was detected in the CYGB‐overexpressing HCT116 (A) and SW620 (C) cells with DCFDA. B, D. ROS production in mitochondria was identified with MitoSOX Red in the HCT116 (B) and SW620 (D) cells. The quantification was determined by flow cytometry. E. Oxygen consumption rate (OCR) of the MOCK‐ and CYGB‐overexpressing HCT116 cells. Respiratory chain inhibitors were serially added to the culture at the indicated time‐points. Basal OCRs are shown by subtracting the rotenone/antimycin‐treated value from the initial value. F. Mitochondrial membrane potential was evaluated by fluorescence staining of mitochondria with the JC‐1 dye. Data represent the mean ± SD of three biological replicates. *P < 0.05, **P < 0.01

CYGB was suggested to play a role in regulating the cellular oxidative status; thus, we first detected the cellular ROS level. As indicated in Figure 2A and C, an obviously elevated ROS level was observed in both colon cancer cell lines that overexpressed CYGB. Moreover, ROS in the mitochondrion was also significantly increased in the CYGB‐overexpressing cells (Figure 2B and D). Furthermore, to determine whether CYGB affects the mitochondrial function, live monitoring using an extracellular flux analyser was performed. Compared with the MOCK cells, the oxygen consumption rate (OCR) decreased in both CYGB cell lines, and the basal respiration rate and ATP production were obviously decreased (Figure 2E; Figure S1A). The fluorescent staining of mitochondria with the JC‐1 dye revealed that CYGB overexpression significantly decreased the mitochondrial membrane potential (FL2) but had no effect on mitochondrial mass (FL1) (Figure 2F; Figure S1B), which corresponded with the decreased oxygen consumption in the CYGB‐OP cell lines. These results suggest that CYGB promoted ROS production and disrupted mitochondrial function in cancer cells.

### 3.3 CYGB improved lipid oxidation and increased sensitivity to ferroptosis

**FIGURE 3:**
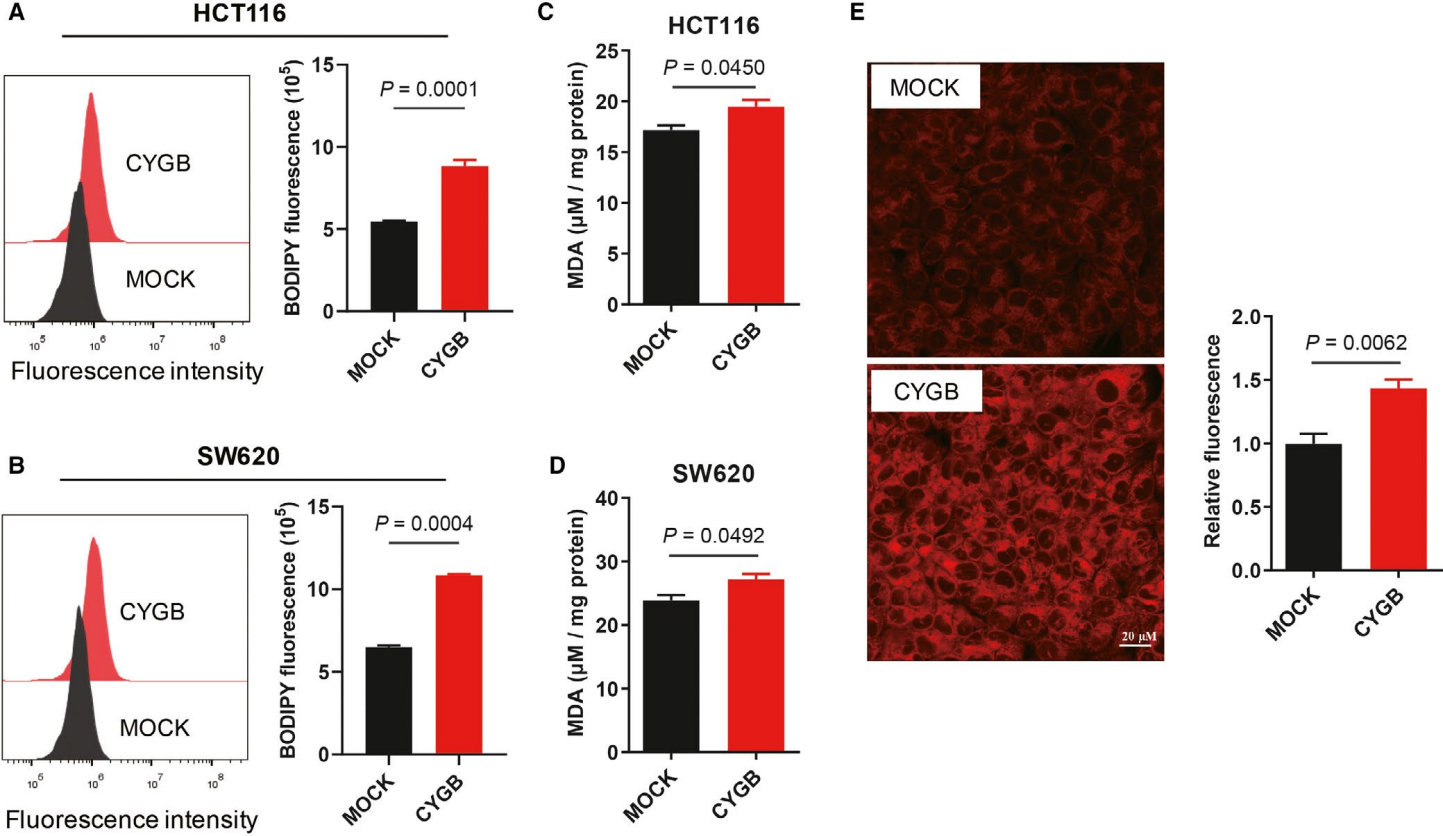
CYGB improved lipid oxidation. A, B. Lipid peroxidation in the MOCK‐ and CYGB‐overexpressing HCT116 (A) and SW620 cells (B) was assessed with C11‐BODIPY by flow cytometry. C, D. Malondialdehyde (MDA) levels were detected using a lipid peroxidation MDA assay kit in the HCT116 (C) and SW620 cells (D). E. Fluorescence staining of cellular iron. The iron was stained with FerroOrange and photographed. The relative fluorescence normalized to the untreated condition was indicated as the quantified data

Increased cellular ROS has been reported to cause lipid peroxidation. As indicated, obviously increased lipid peroxidation was detected in the CYGB‐overexpressing cells measured by the lipid peroxidation probe C11‐BODIPY staining (Figure 3A and B). To further demonstrate lipid peroxidation, MDA, which is a metabolic product of lipid peroxidation, was evaluated in the cells (Figure 3C and D). The increased MDA production strongly indicates increased lipid peroxidation in the CYGB cells. Moreover, an increased level of cellular iron was detected in CYGB‐overexpressing cells (Figure 3E).

**FIGURE 4:**
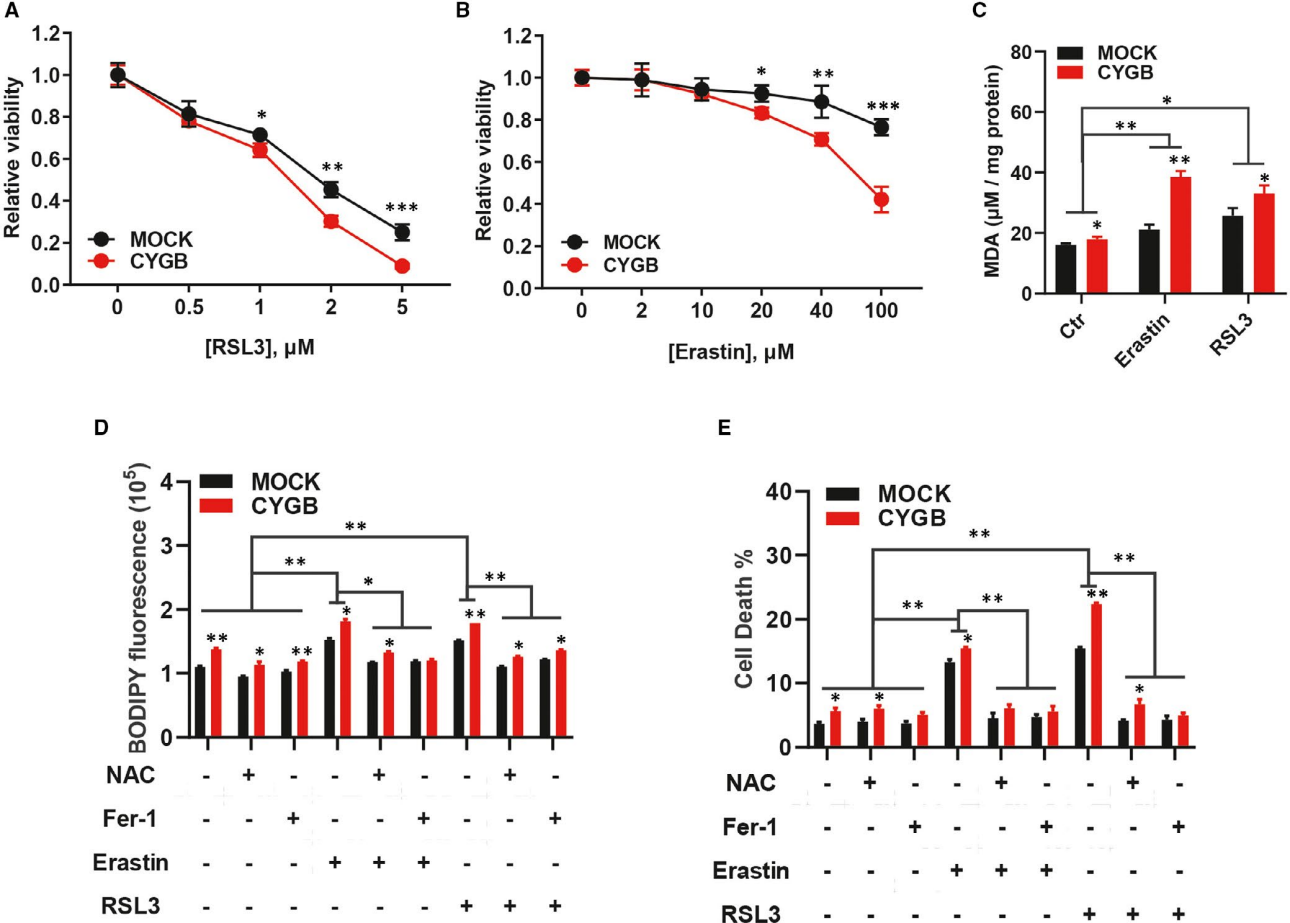
CYGB increased sensitivity to ferroptosis. A, B. Effects of CYGB on cell proliferation after application of the ferroptosis inducers RSL3 (A) or erastin (B). The indicated concentrations of the inducers were applied, and the cell viability was determined after 48 hours. C. Malondialdehyde (MDA) levels were detected in cells with RSL3 or erastin treatment for 24 hours using a lipid peroxidation MDA assay kit. D. Lipid peroxidation was detected after the application of NAC and ferrostatin‐1, with or without RSL3 or erastin. 10 mM NAC and 1 μM Fer‐1 were applied 2 hours before the addition of RSL3 or erastin. An 8 hours treatment of 2 μM RSL3 or 40 μM Erastin was used. E. Cell death was analysed with propidium iodide (PI) staining by flow cytometry after the application of NAC and ferrostatin‐1 (Fer‐1), with or without RSL3 or erastin. 10 mM NAC and 1 μM Fer‐1 were applied 2 hours before the addition of RSL3 or erastin. A 24 hours treatment of 2 μM RSL3 or 40 μM Erastin was used. Data represent the mean ± SD of three biological replicates. *P < 0.05, **P < 0.01

To demonstrate the effects of increased lipid peroxidation, RSL3 and erastin, which are two widely used inducers of ferroptosis, were used. Both RSL3 and erastin inhibited the growth of the HCT116 cells (Figure 4A and B) and SW620 cells (Figure S2A and B). Obviously, the two CYGB‐overexpressing cell lines are more sensitive to cell death induced by RSL3 and erastin. Although an increased cell growth was observed after CYGB siRNAs knock‐down in HCT116 cells (Figure S2C), no significant decreased cell death after treatment with inducers of ferroptosis was detected (Figure S2D). Moreover, there had increased MDA level with the treatment of RSL3 and erastin, while it was more obvious in CYGB‐overexpressing cells (Figure 4C and Figure S2E). Furthermore, the high lipid peroxidation level induced by RSL3 or erastin was significantly decreased in the CYGB‐overexpressing cells with the addition of the ferroptosis inhibitors ferrostatin‐1 and NAC (Figure 4D). Importantly, RSL3‐ or erastin‐induced cell death was also obviously inhibited (Figure 4E), while there had no significant difference with treatment of Z‐VAD, the apoptosis inhibitor (Figure S2F). These results strongly indicated that CYGB was involved in ferroptosis.

### 3.4 CYGB promoted YAP1 expression and was involved in ferroptosis signalling pathway

**FIGURE 5:**
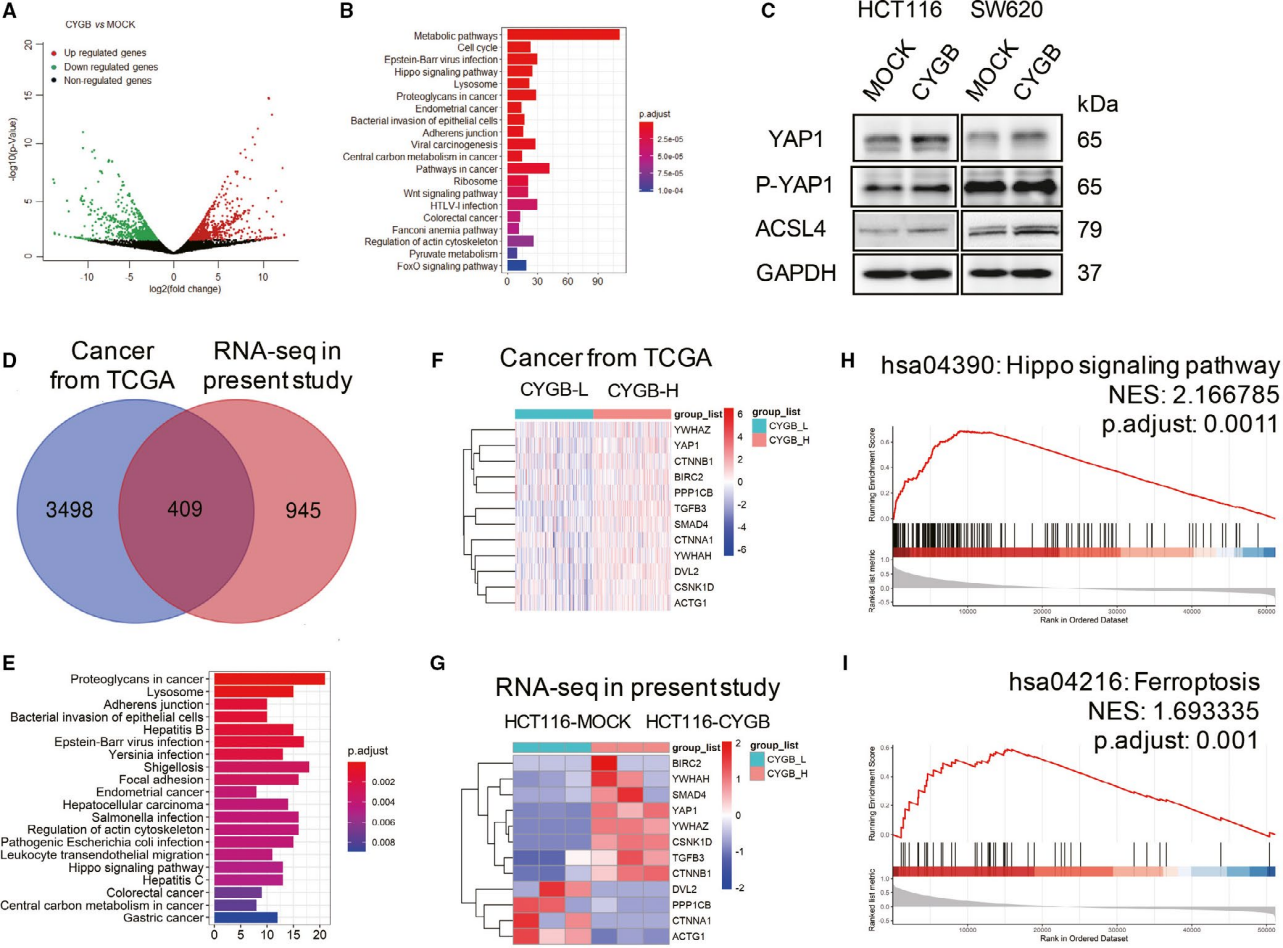
CYGB promoted YAP1 expression and was involved in ferroptosis signalling pathway. A. Demonstration of significant changes in the expression of genes based on RNA‐seq in the CYGB‐overexpressing HCT116 cells compared to MOCK cells. B. The most twenty significantly enriched KEGG pathways based on CYGB overexpression. C. Immunoblotting analysis of YAP1, P‐YAP1 and ACSL4 in the MOCK‐ and CYGB‐overexpressing cells. GAPDH was used as a loading control. D. The comparison of obviously changed genes between RNA‐seq in present study and the colon cancer data from TCGA in Figure 1B. E. The most twenty significantly enriched KEGG pathways based on overlapped genes in D. F, G. The list of obviously changed ferroptosis‐related genes between comparison of CYGB‐low and CYGB‐high expression in colon cancer from TCGA data (F) and RNA‐seq in present study (G). H, I. Gene set enrichment analysis (GSEA) indicates that high expression of CYGB is associated with the hippo (H) and ferroptosis signalling pathway (I) in the TCGA database

We next examined whether a global change was induced by CYGB overexpression through RNA‐seq. A total of 2579 mRNA, including 1356 identified protein coding genes, were significantly changed, with 1336 up‐ and 1243 down‐regulated (Figure 5A). These changes in gene expression indicated alterations throughout the entire cell, and among the top twenty significantly enriched KEGG pathways, metabolic pathways were the most significantly affected by CYGB (Figure 5B). The Hippo signalling pathway was among the top five most significantly enriched KEGG pathways (Figure 5B). As a key factor in Hippo signalling, YAP1 expression and its direct downstream target ACSL4 were verified to be increased (Figure 5C), but YAP1 phosphorylation level was not obviously increased, suggesting that Hippo signalling was not activated by the typical pathway.

To give a further understanding, we compared the common differentially expressed genes between RNA‐seq in present study and genes collected based on the CYGB expression from TCGA (Figure 5D). A total of 409 differentially expressed genes were collected used for KEGG signalling pathway analysis, and the Hippo signalling pathway was listed as one of the significantly enriched KEGG pathways (Figure 5E). The common genes were listed in the data from TCGA (Figure 5F) and RNA‐seq in present study (Figure 5G). Furthermore, gene set enrichment analysis (GSEA) in the TCGA database indicates that high expression of CYGB is associated with the hippo (Figure 5H) and ferroptosis signalling pathway (Figure 5I).

### 3.5 CYGB promoted ferroptosis sensitivity is YAP1 dependent

**FIGURE 6:**
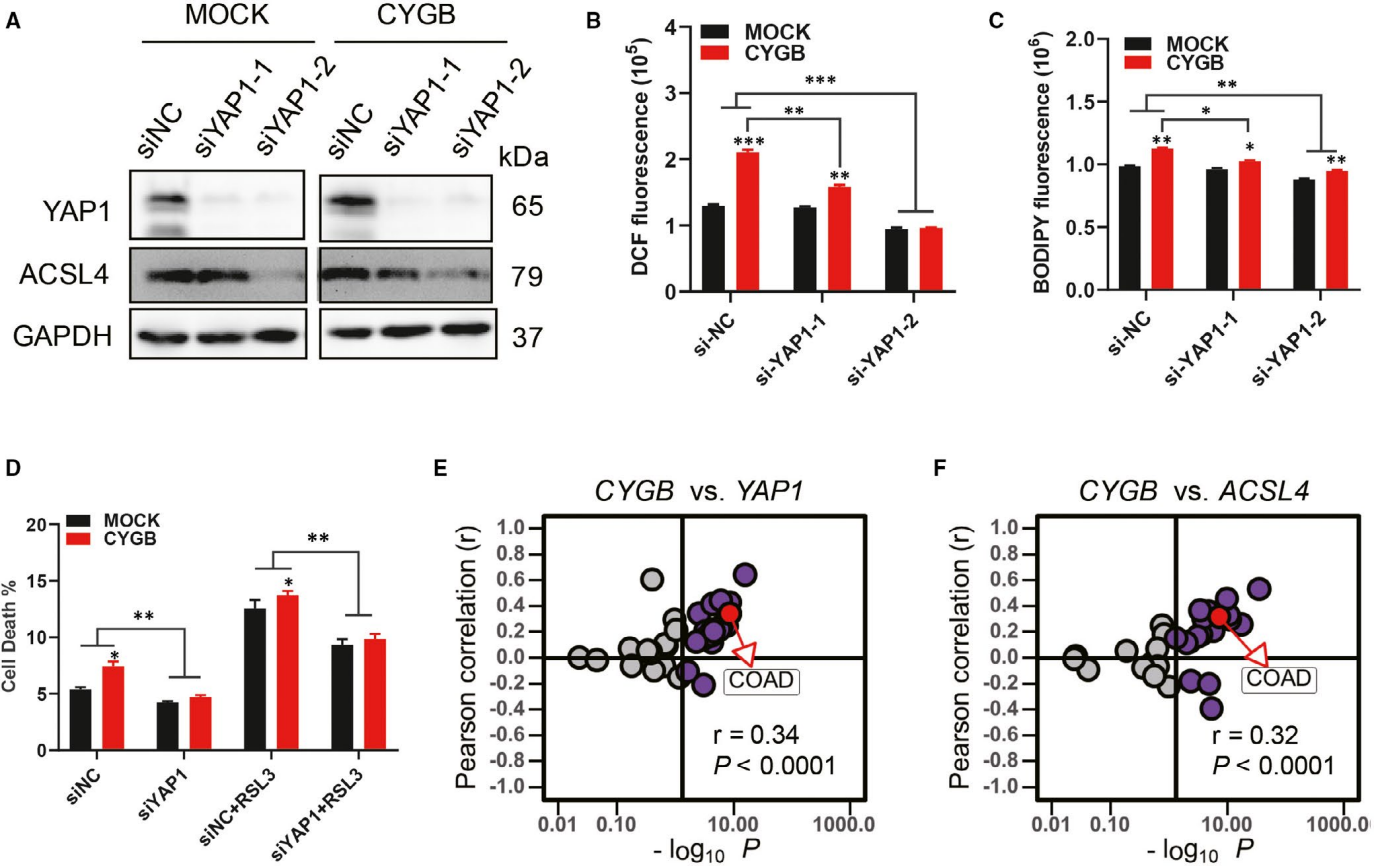
CYGB promoted ferroptosis sensitivity is YAP1 dependent. A. Immunoblotting analysis of YAP1 and ACSL4 in HCT116 cells treated with siRNAs targeting YAP1 for 72 hours. B, C. Cellular (B) and lipid (C) ROS detection with DCFDA and C11‐BODIPY, respectively. D. Cell death analysis with propidium iodide (PI) staining by flow cytometry after the application of RSL3, with or without YAP1 siRNAs. Data represent the mean ± SD of three biological replicates. *P < 0.05, **P < 0.05. E, F. Positive correlations between CYGB and YAP1 (E); and between CYGB and ACSL4 (F) mRNA expression in colon cancer from TCGA

Next, we asked whether the increased sensitivity to ferroptosis in CYGB‐overexpressing cells was related to increased YAP1 expression. Specific siRNAs targeting YAP1 were applied, and the efficiency was confirmed by the obviously decreased levels of YAP1 and its direct downstream target ACSL4 (Figure 6A). As a result of YAP1 knock‐down, the ROS level was decreased in the YAP1 siRNA‐treated cells, and the increased ROS level in the CYGB‐overexpressing cells was recovered to levels similar to those in the control cells (Figure 6B). Moreover, the level of lipid oxidation in the CYGB‐overexpressing cells was also restored (Figure 6C). Importantly, RSL3‐induced cell death was decreased in both the MOCK‐ and CYGB‐overexpressing cell lines with YAP1 knock‐down, and there was no obvious difference between these two cell lines (Figure 6D), indicating that YAP1 was a key factor responsible for RSL3‐induced cell death in the CYGB‐overexpressing cells. To provide further evidence, the colon adenocarcinoma (COAD) samples from TCGA database was utilized, and a significant correlation was observed between *CYGB* and *YAP1* (*r* = 0.34, *P* < 0.0001, Figure 6E) and between *CYGB* and *ACSL4* (*r* = 0.32, *P* < 0.0001, Figure 6F), which was also detected based on the data from Cancer Cell Line Encyclopedia (CCLE) databases (Figure S3A and B). These above results strongly indicated that CYGB was closely correlated with YAP1.

### 3.6 YAP1 was a downstream target of p53

**FIGURE 7:**
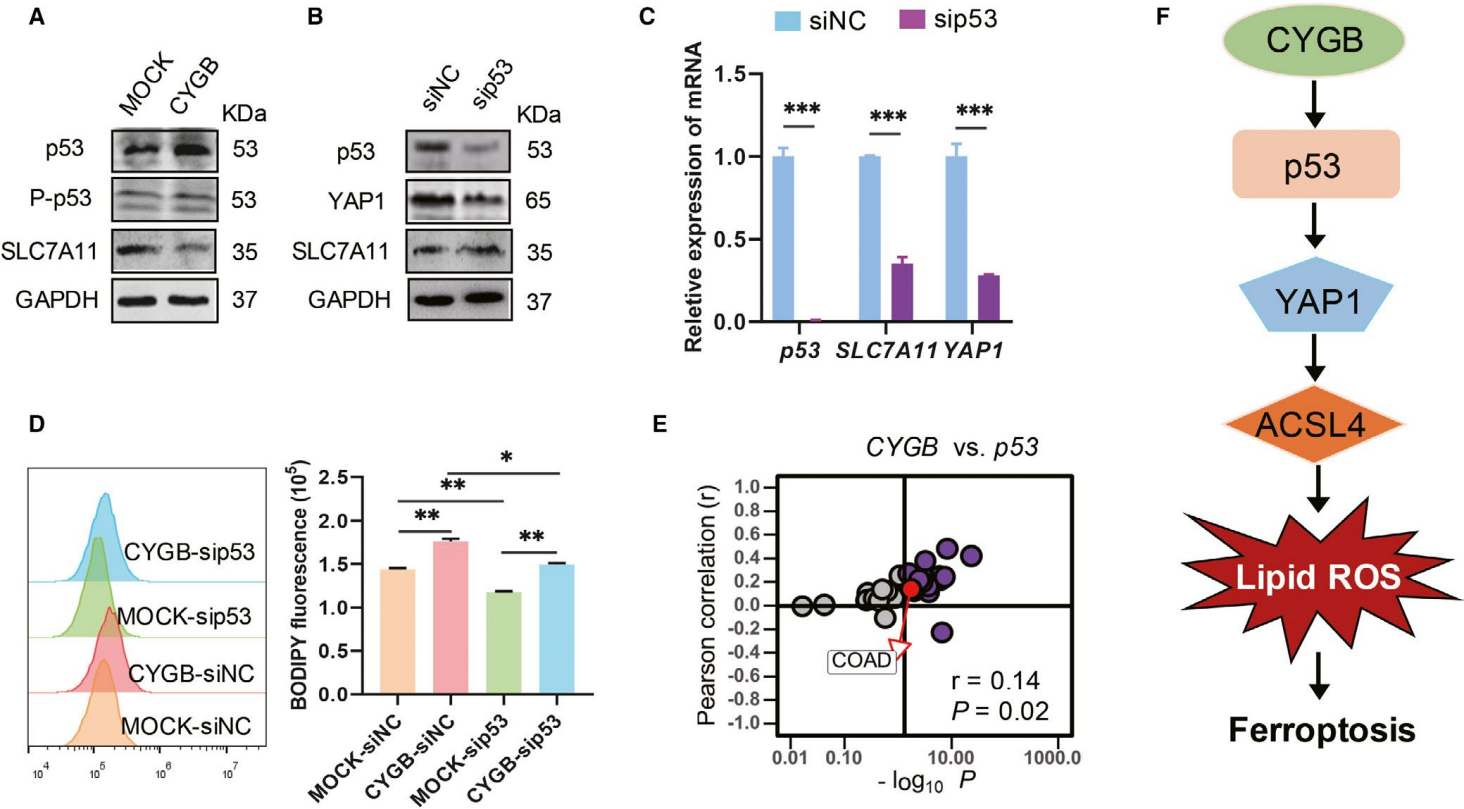
YAP1 was a downstream target of p53. A. Immunoblotting analysis of p53, P‐p53 and SLC7A11 in the MOCK‐ and CYGB‐overexpressing cells. B. Immunoblotting analysis of p53, YAP1 and SLC7A11 in the HCT116 cells treated with siRNAs targeting p53 for 72 hours. C. Transcriptional level of p53, SLC7A11 and YAP1 in the HCT116 cells treated with siRNAs targeting p53 for 72 hours. D. Effects on the lipid peroxidation after p53 knock‐down in the MOCK‐ and CYGB‐overexpressing HCT116 were assessed with C11‐BODIPY by flow cytometry. Data represent the mean ± SD of three biological replicates. *P < 0.05, **P < 0.05. E. Correlations between CYGB and P53 mRNA expression in colon cancer from TCGA. F. A proposed working model of CYGB to promote sensitivity of ferroptosis. YAP1 was the downstream of p53 which was activated by CYGB to promote lipid peroxidation and ferroptosis

Components of the YAP/Hippo and p53 pathways could functionally interact to govern cell fate decisions. So we explored the relation between *YAP1*, *p53* and its direct downstream target SLC7A11 in colon cancer cells. An obviously increased p53 protein expression, but not the phosphorylation level, was identified in CYGB‐overexpressing cells (Figure 7A). After the siRNA knock‐down of *p53*, protein and mRNA levels of *YAP1* were also decreased (Figure 7B). The efficiency of *p53* knock‐down was confirmed and evaluated by detecting *p53* and its downstream target gene *SLC7A11* (Figure 7B and C). Furthermore, the *p53* knock‐down significantly decreased the lipid ROS production in CYGB‐overexpressing cells (Figure 7D). These data indicated the YAP1 was regulated by p53. A further evidence was demonstrated that a significant correlation was observed between *CYGB* and *p53* (*r* = 0.14, *P* = 0.02, Figure 7E).

## 4 DISCUSSION

Colon cancer is one of the most common malignant tumours worldwide and the third leading cause of cancer‐related deaths in both sexes; these deaths are generally due to the late diagnosis and the recurrence or metastasis of tumour cells.^32^, ^33^ Therefore, exploring the key molecules involved in CRC and demonstrating the functional mechanism would provide potential significance for CRC therapy. As a family member of globin, CYGB is considered to act as a tumour suppressor. In the present study, we demonstrated that CYGB inhibited cancer cell growth, decreased mitochondrial function and increased lipid peroxidation. Mechanistically, CYGB‐overexpressing cancer cells were more sensitive to ferroptosis inducers, such as RSL3 and erastin, due to the increase in YAP1 expression.

As a new mode of cell death, ferroptosis is related to the accumulation of lipid peroxidation and is a novel method for cancer cell therapy.^4^, ^34^ There are generally two well‐accepted pathways. One involves the glutamate/cystine antiporter of system xc‐ to import cysteine. The other involves inhibiting the activity of GPX4, which eliminates lipid peroxides. The inactivation of GPX4, an enzyme required for detoxification of lipid hydroperoxides, can induce ferroptosis even when the cellular cysteine and GSH contents are normal.^16^ Many types of cancer cells that are resistant to chemotherapy and certain targeted therapies appear to be sensitive to ferroptosis induced by GPX4 inhibition.^35^, ^36^ As the key component of the Hippo signalling pathway, YAP1 has been reported to regulate multiple biological processes. Although YAP1 is commonly considered a proto‐oncogene,^13^, ^14^, ^15^, ^16^ recent studies have demonstrated its tumour‐suppressive role in haematological cancer, breast cancer and even lung SCC cancer. YAP1 is closely related to ROS. YAP activation led to excessive accumulation of ROS by down‐regulating the antioxidant enzyme GPX2 in a manner related to p63 blockade.^37^ Here, our data also support the positive involvement of YAP in regulating ROS accumulation. However, the accumulation of ROS was closely related to ferroptosis. Generally, mitochondria account for most ROS production. It was reported that ferroptosis was induced due to mitochondrial membrane potential hyperpolarization and lipid peroxide accumulation with cysteine deprivation.^38^ In the present study, the increased ROS production and decreased mitochondrial membrane potential strongly indicated that CYGB disrupted mitochondrial function to contribute to ferroptosis.

Interestingly, the ROS generated by tert‐butyl hydroperoxide (TBH) were reported to induce ferroptosis through p53 activation.^6^ In addition, both p53 and YAP1 were reported to play regulatory roles in the cell cycle and ferroptosis. Components of the YAP/Hippo and p53 pathways functionally and physically interact to govern cell fate decisions.^39^ It was reported that p53 and YAP1 can be dynamically coregulated. YAP1 can directly bind to the TP53 gene promoter and up‐regulate p53 expression, leading to apoptosis during hepatocellular carcinoma chemotherapy. In turn, p53 can bind to the YAP1 promoter and up‐regulate its expression, establishing a positive feedback loop.^40^ Here, we demonstrated that YAP1 was a downstream of p53 and the down‐regulated YAP1 expression was observed in p53 knock‐down cells. The significantly decreased lipid ROS in CYGB overexpressed cells with p53 knock‐down strongly indicated that p53 was in the downstream of CYGB to regulate cellular function. On the other hand, it was reported that the anticancer chemical reagent chaetocin increased ROS, which induced YAP expression in glioma cells independently of the canonical Hippo pathway. Thus, the decreased ROS level due to transfection with YAP1 siRNAs also suggested that there was positive feedback regulation between ROS accumulation and YAP1 expression.

Although CYGB was reported to protect cells against oxidative stress, and the haem of Cygb was suggested to possess a radical scavenging function, no difference was observed in the expression levels of principal antioxidant enzymes, such as haem oxygenase‐1, catalase and superoxide dismutase types 1 and 2, in kidney cortex samples from *Cygb*‐transgenic rats and wild‐type littermates.^41^ Even when CYGB was discovered, there were obviously increased ROS levels and CYGB expression in activated HSCs compared with quiescent cells. Furthermore, CYGB could also be increased under oxidative stress conditions, such as H_2_O_2_ treatment. These results suggested that CYGB could be regulated by ROS and could regulate cellular biological processes. Thus, we investigated the function of constitutively overexpressed CYGB in CRC cells. Although an accumulation of ROS was confirmed, there were no obvious changes in antioxidant enzymes, such as Ho‐1 or catalase (data not shown), between the CYGB‐overexpressing and control cells, and YAP1 was identified as a target of CYGB based on the RNA sequence. Importantly, we observed that the ROS levels were decreased by YAP1 knock‐down. Thus, the accumulation of ROS in CYGB‐overexpressing cells is the result of YAP1 activation, and our results are the first to identify a direct downstream target of CYGB in regulating ROS homeostasis. Furthermore, CYGB could interact with cellular membrane lipids to play a role of cell signalling regulation. It was considered that CYGB could regulate lipid metabolism under oxidative conditions. For example, it could enhance lipid peroxidation, one of the major pathways that produce reactive products and which was also the key step in the ferroptosis. Moreover, the improved expression of CYGB was accompanied by the increased ROS level during the activation of hepatic stellate cells. Based on these above reports and our experimental results, it was conceivable to expect that CYGB owned similar function to enhance lipid peroxidation and contribute to ferroptosis even in different cancers.

In summary, we demonstrated that CYGB‐overexpressing cells promote sensitivity to ferroptosis due to lipid peroxidation accumulation. YAP1 was a key downstream target of CYGB through p53 regulation to promote ferroptotic cell death signalling pathway (Figure 7F). Thus, our findings emphasize the importance of CYGB in ferroptosis regulation in colon cancer cells.

## CONFLICT OF INTEREST

The authors declare no conflict of interest.

## AUTHOR CONTRIBUTIONS

Yang Xi, Shazhou Ye and Mingjun Xu designed the study. Shazhou Ye, Mingjun Xu, Tingwei Zhu, Jiayi Chen, Haizhong Jiang and Qingfang Zheng performed the experiments. Shazhou Ye, Shanping Shi and Qi Liao carried out the TCGA data analysis. Yang Xi and Xiaoyun Ding discussed the research and edited the manuscript. Yang Xi supervised the research. All authors read and approved the final publication.

## Supporting information

Figure S1‐S3
