# Supplementary material for: Cytoglobin promotes sensitivity to ferroptosis by regulating p53‐YAP1 axis in colon cancer cells
Source: J Cell Mol Med. 2021 Feb 21;25(7):3300–11. doi: 10.1111/jcmm.16400 (PMC8034452; doi:10.1111/jcmm.16400)
Supplement: Supplementary file 1 — Figure S1‐S3 [file JCMM-25-3300-s001.pdf]

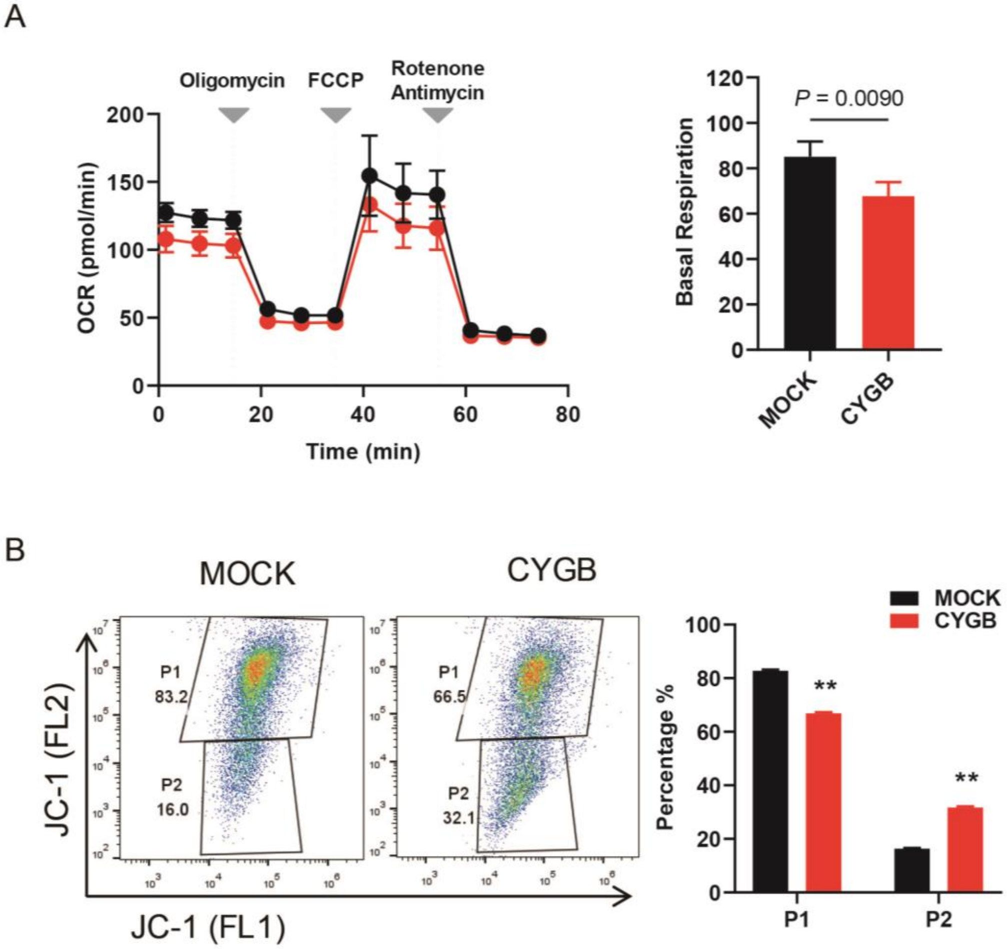

Figure S1. CYGB disrupted mitochondrial function

A. Oxygen consumption rate (OCR) of the MOCK- and CYGB-overexpressing SW620 cells. Respiratory chain inhibitors were serially added to the culture at the indicated time points. Basal OCRs are shown by subtracting the rotenone/antimycin-treated value from the initial value. **B.** Mitochondrial membrane potential was evaluated by fluorescence staining of mitochondria with the JC-1 dye. Data represent the mean ± S.D. of three biological replicates. *P < 0.05, **P < 0.01.

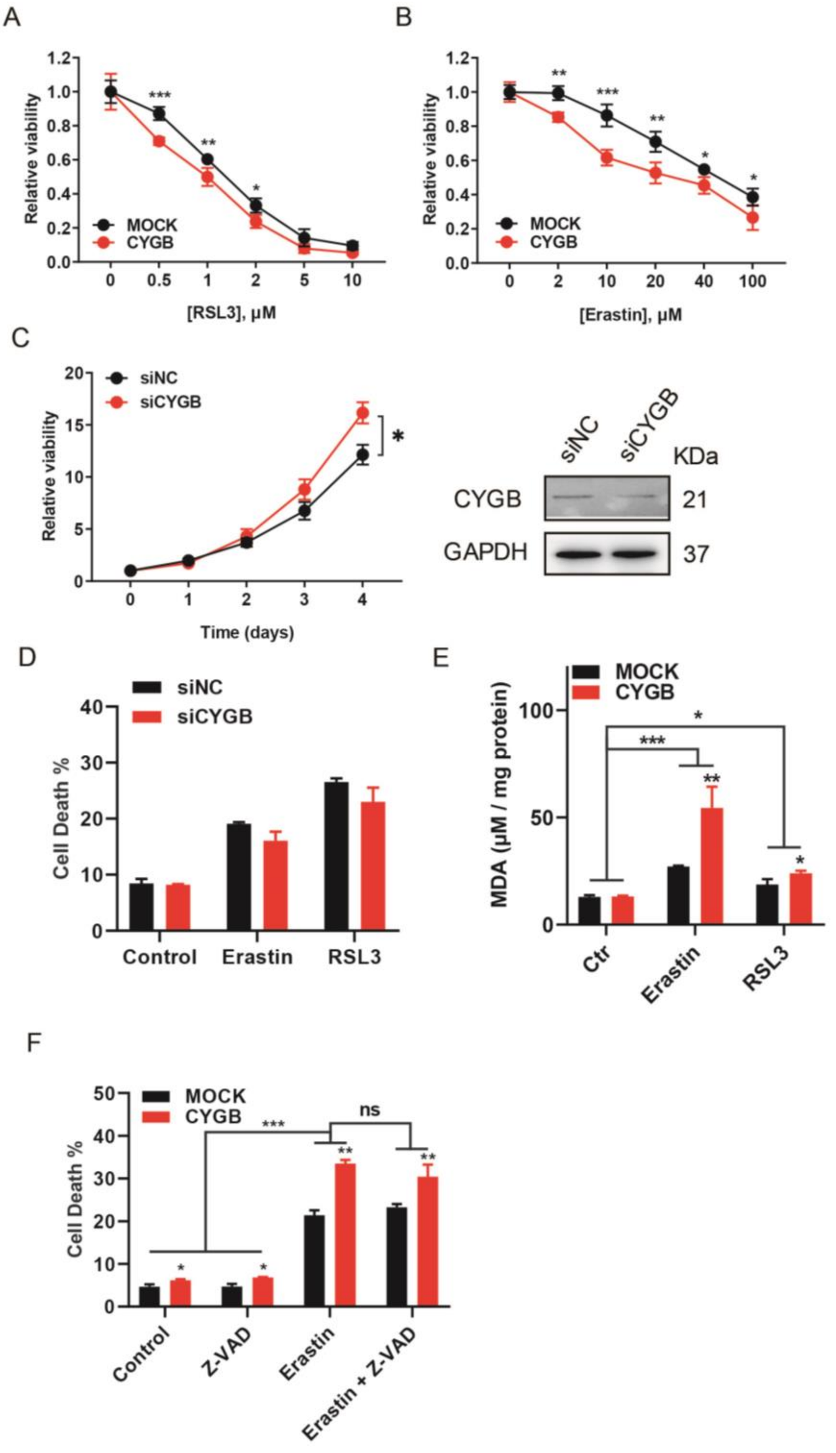

Figure S2. CYGB increased sensitivity to ferroptosis

A, B. Effects of CYGB on cell proliferation after application of the ferroptosis inducers RSL3 (**A**) or erastin (**B**). The indicated concentrations of the inducers were applied, and the cell growth was determined after 48 hours. Relative viability normalized to the untreated condition was used. Data represent the mean ± S.D. of three biological replicates. **P* < 0.05, ***P* < 0.01, and ****P* < 0.001. **C.** Effects of CYGB knockdown on cell proliferation after CYGB siRNAs (siRNA of CYGB 5′-GGA GGA AUC CCU GAC UCA A-3′) mediated knockdown in HCT116 cells. The knockdown efficiency was verified by immunoblotting. **D**. Effects of CYGB knockdown on cell death after the application of ferroptosis inducers RSL3 or erastin. Cell death detection was applied with a 24-hour treatment of ferroptosis inducers RSL3 or erastin after 72 hours CYGB knockdown. **E.** Malondialdehyde (MDA) levels were detected in cells with RSL3 or erastin treatment using a lipid peroxidation MDA assay kit. **F.** Cell death was analyzed with propidium iodide (PI) staining by flow cytometry after the application of Z-VAD, with or without erastin.

A B

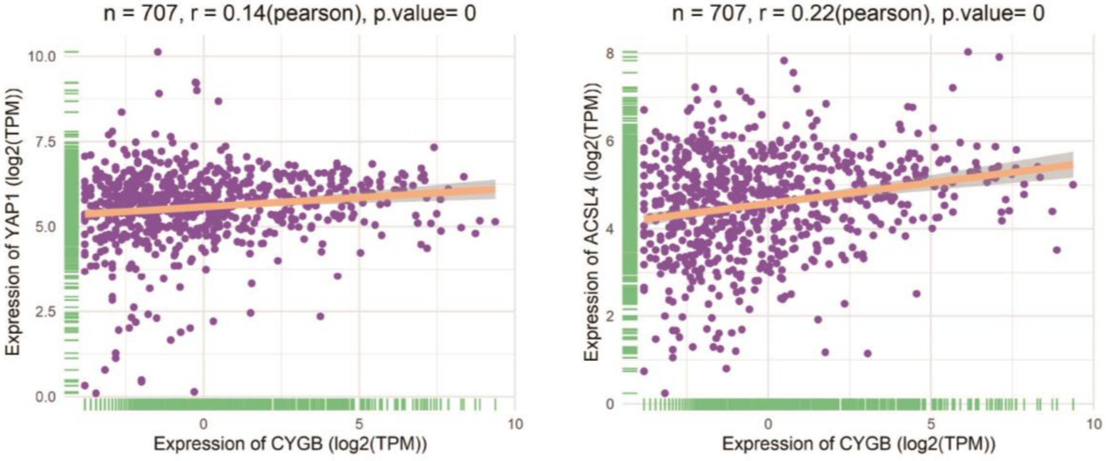

Figure S3. CYGB had the significant correlation with YAP1
A, B. Positive correlations between CYGB and YAP1 (A); and between CYGB and ACSL4 (B) mRNA expression in colon cancer cells from Cancer Cell Line Encyclopedia (CCLE) databases.
